# Physical health policies and metabolic screening in mental health care systems of sub-Saharan African countries: a systematic review

**DOI:** 10.1186/s13033-017-0141-7

**Published:** 2017-04-19

**Authors:** James Mugisha, Marc De Hert, Brendon Stubbs, David Basangwa, Davy Vancampfort

**Affiliations:** 1 Butabika National Referral and Mental Health Hospital, Kampala, Uganda; 2 Kyambogo University, Kampala, Uganda; 3 KU Leuven-University of Leuven, University Psychiatric Center KU Leuven, Louvain-Kortenberg, Belgium; 4 Physiotherapy Department, South London and Maudsley NHS Foundation Trust, London, UK; 5 Health Service and Population Research Department, Institute of Psychiatry, Psychology and Neuroscience, King’s College London, De Crespigny Park, London, UK; 6 Department of Rehabilitation Sciences, KU Leuven-University of Leuven, Louvain, Belgium

**Keywords:** Physical health, Somatic, Metabolic, Mental health, Sub-Sahara Africa

## Abstract

**Background:**

There is a need for interventions to address the escalating mental health burden in sub-Saharan Africa (SSA). Addressing physical health needs should have a central role in reducing the burden and facilitating recovery in people with severe mental illness (SMI). We systematically investigated (1) physical health policies in the current mental health plans, and (2) the routine metabolic screening rates for people with SMI in SSA.

**Methods:**

The Mental Health Atlas and MiNDbank of the World Health Organization were screened for physical health policies in mental health plans. Next, we systematically searched PubMed from inception until February 1st, 2017 for relevant studies on metabolic screening rates in people with SMI in SSA.

**Results:**

The current systematic review shows that in 22 screened plans only 6 made reference to a physical health component or policy. Only the South-African mental health plan reported about routine screening and treatment of physical illness for people with SMI. In 2 South-African studies (n = 431) routine screening was unacceptably low with less than 1% adequately screened for all modifiable metabolic syndrome risk factors.

**Conclusions:**

Our review data clearly show that a physical health policy is yet to be embraced in mental health care systems of most SSA countries. There is a clear need for integrated mental and medical services in SSA. All psychiatric services, including poorly developed community-based primary health care settings should standardly assess the body mass index and waist circumference at initiation of psycho-pharmacotherapy, and afterwards at regular intervals. Optimal monitoring should include assessments of fasting glucose, lipids, cholesterol, and blood pressure. Mental health care providers in SSA countries need to be informed that their roles extend beyond taking care of the mental health of their patients and assume responsibility for the physical health of their patients as well. Policy makers should be made aware that investment in continued medial education and in screening for physical health risks could optimize mental and physical health improvements. The increased physical health needs of people with mental illness should be integrated into the existing Information, Education and Communication public health awareness programs of the World Health Organization.

## Background

Mental and substance use disorders are the leading cause of years lived with disability in sub-Saharan Africa (SSA), accounting for almost 20% of all disability-associated burden [1]. The burden is estimated to increase even further [2]. The consequences of this rising burden will be devastating and will be worsened by secondary physical health co-morbidities people with mental and substance use disorders are confronted with [3–5]. Combined mental and physical health problems will have in particular large consequences for women as in SSA women are mostly generating the family income. They mostly rely for this on labor-demanding jobs in the informal sector with no job security or compensation for lost income. Maintaining physical health is therefore crucial for their livelihoods and the society as a whole.

To date, a mental health policy has been relatively low on the priority list in most of SSA countries [6]. In most countries still less than 1% of the health budget is spent on mental health [7]. As a result mental health services are poorly resourced and treatment rates for people with mental disorders remain low, with treatment gaps over 90% [8]. Pharmacotherapy, and in particular antipsychotic medication use, is also the mainstay of therapy programs in SSA [6], but has important cardio-metabolic risk factors [9, 10]. The metabolic risk associated with antipsychotics has led to the development of several International treatment guidelines for routine metabolic screening and monitoring in patients taking antipsychotic medication [11, 12]. The extent and frequency of screening is debatable, but there is global consensus that baseline metabolic screening should worldwide form part of the standard package of care for all patients with a regular prescription of any antipsychotic [13]. Several years ago reports of routine metabolic screening practices in large cohorts of patients from high-income countries (USA, UK, Australia, Canada and Spain) have shown that metabolic testing was not optimal despite widespread dissemination of the guidelines and a high level of awareness of the metabolic risks associated with antipsychotic medication use among psychiatrists [14]. Recent reports show that progress has been made [15], albeit inconsistently [16] in these high-income countries. To date, metabolic screening rates in people under antipsychotic treatment in middle-to-low-income countries have however not been explored systematically.

The aim of the current systematic review is twofold. First, we explored whether or not within the current mental health plans of SSA countries a physical health component or policy has been included. Second, we systematically investigated the routine metabolic screening rates for people with severe mental illness in SSA countries.

## Methods

### Screening for physical health components or policies in mental health plans in sub-Saharan Africa

In a first stage, we screened the latest Mental Health Atlas [17]. If the country data were not available in the latest edition, the penultimate edition was screened. With data from 171 World Health Organization (WHO) Member States, the Mental Health Atlas provides a comprehensive overview of mental health plans worldwide. Data abstracted were the presence of a mental health plan (yes or no).

In a second stage, if a mental health plan was available the full-text documents were retrieved via the MiNDbank of the World Health Organization [18]. Policies written in Swahili, English, French, Spanish or Portuguese were evaluated. If the mental health policy and/or plan was not available, google scholar was screened using the search terms: “mental health” AND “plan” OR “policy” and the name of the country, or its equivalents in other languages. Mental health plans were screened for addressing physical health needs in their mental health policies. Search terms used, were: “physical” OR “somatic” or its equivalents in other languages.

In a third stage, we summarized the physical health components/policies in the mental health plans.

### Identification of studies on metabolic screening conducted in people under antipsychotic treatment in SSA

#### Search strategy

We systematically search PubMed from inception until February 1st, 2017 for relevant studies on metabolic screening rates in people with severe mental illness under antipsychotic treatment in SSA. The following search strategy was used: “screen*” or “monitor*” or “exam*” or “assessment” AND “weight” or “obesity” or “metabolic” or “glucose” or “diabetes” or “lipids” or “cholesterol” AND “mental” or “depression” or “psychosis” or “schizophrenia” or “bipolar” or “antipsychotics” AND the name of the country.

#### Eligibility criteria

##### Participants

Studies examining routine metabolic screening practices for severe mental ill patients under psychiatric care who were prescribed antipsychotics were included. We did not exclude any people due to age or whether or not they were diagnosed with Statistical Manual [19, 20] or International Classification of Disease [21] criteria or with validated diagnostic tools.

##### Outcome measure

The primary outcome measure was any routine metabolic screening rate (prevalence) in any mental health setting in SSA. If available also the routine metabolic screening rate for controls without mental illness were included.

##### Study design

There was no limitation regarding study design. For longitudinal or intervention studies routine baseline data were included.

#### Exclusion criteria

In case of overlap only the most recent study or the study with the largest dataset were included. No additional exclusion criteria were applied.

#### Study selection

Two reviewers (DV and BS) screened titles and abstracts of all potentially eligible articles. Both authors applied eligibility criteria, and a list of relevant studies was developed. When necessary, the protocol stated that the corresponding author of a study would be contacted up to two times in a 4-week period to request data that would enable inclusion in the current analyses.

#### Data extraction

Two authors (DV, BS) extracted data using a predetermined data extraction form. The data extracted were country, study setting (inpatients versus outpatients versus community patients), patient’ characteristics (diagnosis, age, % male) and the metabolic screening rates (%) for patients and if available controls.

## Results

### Physical health components or policies in mental health plans in sub-Saharan Africa

In terms of mental health policies, 69% (=33/48) of SSA countries report having a mental health plan. Ten full-texts of the mental health plans were not found while one (Sudan) was written in Arabic, and therefore not meeting our inclusion criteria. Six of 22 screened mental health plans included a physical health component or policy. An overview of the presence of a mental health plan and the presence of a physical health component/policy in these plans is presented in Table 1. The physical health components/policies in the mental health plans are prescribed in detail in Table 2. Briefly, while the mental health plan of Burundi reported only that there should also be a focus on the physical well-being of people with mental illness and the mental health plans of Ethiopia, Ghana, Nigeria and Zambia stated that improved access to care and treatment of co-morbid physical conditions should be an aim, the mental health plan of South-Africa was the only more detailed one. In this plan it was stated that the aim should be to implement a routine screening and treatment of physical illness in all consultations for people with mental illness.

**Table 1:** Overview of the presence of a mental health plan, a physical health component/policy and metabolic screening research results in sub-Saharan African countries (n = 48)

Country	Official mental health plan	A physical health component/policy	PubMed search results (potential relevant/obtained)
Angola (2011)	Yes	No	0/9
Benin (2014)	No	–	0/58
Botswana (2014)	Yes	No	0/37
Burkina Faso (2014)	Yes	No	0/19
Burundi (2014)	Yes	Yes	0/14
Cameroon (2011)	No	–	0/48
Cape Verde (2011)	Yes	No	0/6
Central African Republic (2014)	Yes	No	0/4
Chad (2011)	Yes	NA	0/36
Comoros (2011)	Yes	NA	0/5
Congo (2014)	No	–	0/70
Côte d’Ivoire (2014)	Yes	No	0/21
Democratic Rep. of the Congo (2011)	Yes	No	0/30
Djibouti (2014)	No	–	0/6
Equatorial Guinea (2014)	No	–	0/0
Eritrea (2011)	No	–	0/17
Ethiopia (2014)	Yes	Yes	0/238
Gabon (2011)	No	–	0/1
Gambia (2014)	Yes	No	0/19
Ghana (2014)	Yes	Yes	1/121
Guinea (2014)	Yes	NA	0/3161
Guinea-Bissau (2011)	No	–	0/2
Kenya (2011)	Yes	No	1/180
Lesotho (2014)	No	–	0/6
Liberia (2014)	Yes	No	0/10
Madagascar (2014)	Yes	No	0/12
Malawi (2014)	Yes	No	0/46
Mauritania (2011)	Yes	NA	0/7
Mauritius (2014)	No	–	0/16
Mozambique (2014)	Yes	No	0/12
Namibia (2014)	Yes	No	0/12
Niger (2011)	Yes	NA	0/90
Nigeria (2014)	Yes	Yes	0/816
Rwanda (2014)	Yes	No	0/56
São Tomé and Príncipe (2014)	Yes	NA	0/0
Senegal (2014)	No	–	0/46
Seychelles (2014)	No	–	0/8
Sierra Leone (2014)	Yes	No	0/14
Somalia (2014)	No	–	0/50
South-Africa (2014)	Yes	Yes	3/1505
South-Sudan (2014)	No	–	0/7
Sudan (2011)	Yes	Not checked^a^	0/86
Swaziland (2014)	No	–	0/12
Togo (2014)	Yes	NA	0/54
Uganda (2014)	Yes	No	0/266
United Republic of Tanzania (2011)	Yes	NA	0/116
Zambia (2014)	Yes	Yes	0/41
Zimbabwe (2014)	Yes	NA	0/99
Summary	69% (33/48) has an official mental health policy	27% (6/22) described a physical health component/policy	5/7439

*NA* not available

^a^Plan written in Arabic

**Table 2:** Physical health components/policies in mental health plans of sub-Saharan African Countries

Country	Physical health components/policies
Burundi	The focus should also be on the physical well-being
Ethiopia	Providing physical health care to persons with mental disorders is needed. By integrating mental health services into the primary health care system it is envisioned that those with both physical and mental health related needs will be treated in a seamless and comprehensive manner
Ghana	The Mental Health Authority should ensure there is implementation of legislation to protect the mentally ill with regards to employment, accommodation and access to treatment which should include access to physical health care treatment for those who cannot afford to be part of the National Health Insurance Scheme
Nigeria	People with mental illness have a higher premature mortality than the general population from physical illness. It is therefore extremely important to ensure adequate physical health care and health promotion to people with mental illness, particularly those being looked after in psychiatric units and hospitals
South-Africa	Mental and substance use disorders are closely correlated with physical diseases including both communicable diseases such as HIV and AIDS and non-communicable diseases such as heart disease and cancer. The aim is to implement a routine screening and treatment of physical illness in all consultations for people with mental illness
Zambia	Improved access to care and treatment of co-morbid physical conditions is an aim

### Routine metabolic screening studies conducted in people under antipsychotic treatment in SSA

#### Search results

Out of 7439 search hits, 5 potentially eligible studies were retrieved. After applying the eligibility criteria 2 studies [22, 23] were included. An overview of the search results for each country is presented in Table 1. Reason for exclusion was lack of routine metabolic screening (n = 3).

#### Participants and study characteristics

Details of the participants and study characteristics are presented in Table 3. In total 431 patients with severe mental illness of which 206 men (47.8%) were included. The mean age was 35.2 [23] and 42.9 [22] years. Both studies were cross-sectional and performed in South-Africa. While one study [23] was executed in outpatients, the other one [22] was done in community patients.

**Table 3:** Metabolic screening in people with severe mental illness in sub-Saharan Africa

First author	Country	Design	Participants	Metabolic screening rates patients	Metabolic screening rates controls
Saloojee et al. [23]	South-Africa	Cross-sectional	331 (167♂) outpatients with SMI; 35.2 ± 12.0 years	0.6% waist circumference	–
				3.9% fasting glucose	
				1.8% fasting lipids (triglycerides, HDL-cholesterol)	
				99% blood pressure	
Ludwick and Oosthuizen [22]	South-Africa	Cross-sectional	100 (39♂) community patients with SMI; 42.9 ± 11.1 years	25.7% weight	68% weight
				0% waist circumference	86% blood pressure
				38.6% blood pressure	

*SMI* severe mental illness

#### Metabolic screening outcomes

Details of the metabolic screening rates are presented in Table 3. In both studies, waist circumference was measured in less than 1% of the studies.

## Discussion

The current systematic review shows that in those SSA countries that have a mental health plan (n = 33, 70%) only 6 were found to make reference to a physical health component or policy. Only the South-African mental health plan stated that a routine screening and treatment of physical illness should be implemented in all consultations for people with mental illness. Our review findings clearly indicate that although physical health concerns are becoming acknowledged as an important focus in the multidisciplinary management of severe mental health problems [24], a clear physical health policy is yet to be embraced in mental health care systems of most SSA countries. The lack of priority given to physical health concerns is also mirrored in the limited number of studies exploring the metabolic screening rates in the management of mental health problems. Only 2 South-African studies assessed the metabolic screening rates. In both studies, routine screening was unacceptably low with less than one percent adequately screened for waist circumference, a modifiable metabolic syndrome risk factor for type 2 diabetes mellitus and cardiovascular disease [25]. This is surprising as waist circumference is very easy to measure and inexpensive.

Hence, there is a clear need to re-orient the current mental health care systems in SSA and focus more on the physical health needs of people with mental illness. An important environmental reason is the lack of integrated mental and medical services locally and the poorly developed community-based psychiatric services resulting in an overreliance on institutional care [26]. Closer integration of primary care and mental health in SSA countries is needed. Many mental health providers do not ask about medical issues or test for them because of lack of consideration of this health care aspect, lack of time or lack of resources directly available to them. For example, in the study of Ludwick and Oosthuizen [22] 90% of the mental health providers believe that patients with psychiatric disorders are not being discriminated against and that they are being monitored as regularly as or even more than the rest of the clinical population. The prejudice may therefore not be conscious, but on an unconscious level, which makes it even more complex to address [22]. Lack of knowledge is also exemplified in the observation that only 10% of mental health providers are aware of the fact that people with severe mental illness die earlier than the general population [22].

Therefore, first of all, there is a clear need to increase awareness of the importance physical health needs of patients with severe mental illness among mental health providers in SSA. Continued medical education (CMEs which is a common practice in SSA) [6] should be used to inform these mental health care providers on the importance of assessing physical health risks in people with mental illness. Mental health care providers in low-and-middle-income countries need to be informed that their roles extend beyond taking care of the mental health of their patients and assume responsibility for the physical health of their patients as well. Policy makers should be made aware that investment in CME and in screening for physical health risks could optimize mental and physical health improvements. However, effective monitoring of metabolic risks is not sufficient on its own, as appropriate treatment is also mandatory [27].

Secondly, governmental and non-governmental agencies in the region will do well to increase public health awareness of the increased physical health risks of people with severe mental illness.

For example, the increased physical health needs should be integrated into the existing Information, Education and Communication (IEC especially on mental health and lifestyle diseases) public health awareness programs of the World Health Organization [28]. Targeted and regular messages should be developed in order to make these campaigns affordable in SSA countries. At a minimum, the physical health risks and the benefits of regular physical health screening should be properly outlined. Action is urgently needed. While the worldwide disability-associated burden and premature mortality due to type 2 diabetes mellitus and cardiovascular diseases are projected to increase by 2020, the largest increases in deaths are predicted for SSA countries [29]. SSA is experiencing the most rapid urbanization worldwide which may be contributing to more sedentary behaviours and a rising prevalence of non-communicable diseases [30]. Against this background, people with severe mental illness in SSA, especially those under antipsychotic treatment, are at particularly high risk. Without adequate screening the increasing risks will, in all likelihood, go undetected in the overwhelming majority of patients with severe mental illness. We advocate that all psychiatric services in SSA, even the lowest-resourced one should assess at a minimum the body mass index and waist circumference at initiation of pharmacotherapy and afterwards at regular intervals. Optimal monitoring should also include assessments of fasting glucose, lipids, cholesterol, and blood pressure.

Future research should explore whether complying with the minimum and optimal standards and, if needed, treatment will reduce the risk for future type 2 diabetes and cardiovascular diseases, YLD and consequently disability-associated burden. In order to justify the inclusion of more costly fasting blood screening as a routine component, cost-benefit analysis are required in order to determine and quantify the financial implications of diverting resources or investing funds into such initiatives. Such economic rationales must aim to include cost-savings associated with prevention in the context of the treatment of physical health comorbidities and ideally potential benefits regarding preventing future episodes of poor mental health. Finally, future research in SSA on behavioral health should incorporate the mental health and physical health of people with mental illness rather than the current trend of focusing research solely on one or the other.

In conclusion, the current data shows that in SSA the physical health risks is largely ignored in the current mental health policies in most SSA countries. Policy makers and existing work force should be informed about the importance of considering physical health needs in the multidisciplinary treatment of people with mental health problems.

## Abbreviation

SSA: sub-Saharan Africa
